# Physicians’ preferences for bone metastases treatments in France, Germany and the United Kingdom

**DOI:** 10.1186/s12913-018-3272-x

**Published:** 2018-07-03

**Authors:** Yi Qian, Jorge Arellano, Francesca Gatta, Guy Hechmati, A. Brett Hauber, Ateesha F. Mohamed, Amit Bahl, Roger von Moos, Jean-Jacques Body

**Affiliations:** 1 Health Economics, Amgen Inc, Thousand Oaks, CA 91320 USA; 2 Health Economics, Amgen (Europe) GmbH, Zug, Switzerland; 3 RTI Health Solutions, Research Triangle Park, NC USA; 4 Bristol Haematology and Oncology Centre, University Hospitals Bristol, Bristol Royal Infirmary, Avon, Bristol, UK; 5 Department of Oncology/Hematology, Kantonsspital Graubünden, Chur, Switzerland; 6 Department of Medicine, CHU Brugmann, ULB, Brussels, Belgium

**Keywords:** Discrete-choice experiment, Bone metastases, Bone-targeted agents, Preference, Skeletal-related event, Bone pain

## Abstract

**Background:**

Several bone-targeted agents (BTAs) are available for preventing skeletal-related events (SREs), but they vary in terms of efficacy, safety and mode of administration. This study assessed data on European physicians’ treatment preferences for preventing SREs in patients with bone metastases from solid tumours.

**Methods:**

Physicians completed a web-based discrete-choice experiment survey of 10 choices between pairs of profiles of hypothetical BTAs for a putative patient. Each profile included five attributes within a pre-defined range (primarily based on existing BTAs’ prescribing information): time (months) until the first SRE; time (months) until worsening of pain; annual risk of osteonecrosis of the jaw (ONJ); annual risk of renal impairment; and mode of administration. Choice questions were developed using an experimental design with known statistical properties. A separate main-effects random parameters logit model was estimated for each country and provided the relative preference for the treatment attributes in the study.

**Results:**

A total of 191 physicians in France, 192 physicians in Germany, and 197 physicians in the United Kingdom completed the survey. In France and the United Kingdom, time until the first SRE and risk of renal impairment were the most important attributes; in Germany, time until the first SRE and delay in worsening of pain were the most important. In all countries, a 120-min infusion every 4 weeks was the least preferred mode of administration (*p* < 0.05) and the annual risk of ONJ was judged to be the least important attribute.

**Conclusions:**

When making treatment decisions regarding the choice of BTA, delaying the onset of SREs/worsening of pain and reducing the risk of renal impairment are the primary objectives for physicians.

## Background

Bone is the most common site for metastasis in patients with advanced solid tumours. Approximately 70% of patients with breast cancer, 80–90% of individuals with prostate cancer and 30–40% of those patients with lung, kidney or thyroid cancer will eventually progress to metastatic bone disease [1, 2]. Patients with bone metastases frequently experience bone complications (skeletal-related events, SREs) that are commonly defined as pathologic fracture, spinal cord compression and the requirement for surgery or radiation to bone. SREs cause pain (persistent in the case of pathologic fractures; transient surges when associated with radiation to bone), impair movement and reduce load bearing and functional capacity [1, 3]. Overall, SREs are associated with increased morbidity and mortality and reduced quality of life [4–7]. Surgery to bone and spinal cord compression can result in long, sometimes traumatic, inpatient stays and substantial healthcare costs [8–10]. When considering specifically the economic burden of these events, studies have demonstrated that SREs may result in considerable healthcare resource utilisation, equating to an average cost per SRE in Europe of €13,407–51,188 [11–13]. On average, patients not receiving bone-targeted agents (BTAs) experience approximately 1.5–3.7 SREs a year [8] and up to 64% of patients with bone metastases develop these bone complications [7, 14, 15]. Furthermore, patients are at greater risk of subsequent SREs and have a poorer prognosis after their first event [8, 16].

Several BTAs are approved for use in the European Union (EU) to prevent SREs in patients with bone metastases secondary to solid tumours; the majority of BTAs are bisphosphonates (ibandronate, clodronate, pamidronate and zoledronic acid). Of the bisphosphonates, zoledronic acid is the only agent approved for use in patients with all solid tumours and is considered by many to be the gold standard; indeed, placebo-controlled phase 3 studies show that treatment with zoledronic acid delays time to a SRE by up to 5.6 months in patients with cancer and bone metastases [7, 15]. However, bisphosphonates are excreted by the kidneys and they should be used cautiously in patients with renal dysfunction [17–20]. In addition, zoledronic acid and pamidronate are not recommended for patients with severe renal impairment [18, 19]. Denosumab, a receptor activator of nuclear factor kappa B (RANK) ligand inhibitor, was approved in 2011 in the EU for use in patients with solid tumours and bone metastases, after having demonstrated superiority versus zoledronic acid in patients with prostate cancer, breast cancer and other solid tumours in three large randomised clinical trials [21–24]. An integrated analysis of the trials found that denosumab delayed time until the first SRE by 8.2 months compared with zoledronic acid [23]. Furthermore, in contrast to bisphosphonates, denosumab can be used without dose adjustment in patients with severe renal disease. Both bisphosphonates and denosumab are associated with a risk of osteonecrosis of the jaw (ONJ); in the integrated analysis of zoledronic acid versus denosumab, the incidence was 1.3 and 1.8%, respectively [23]. In addition to preventing SREs, bisphosphonates and denosumab have been shown to improve bone pain related outcomes, with denosumab delaying onset and increases in pain by considerably longer than zoledronic acid [17–20, 25].

Information on how the risks and benefits of BTAs influence prescribing decisions will help us to understand which risk–benefit profiles are acceptable to physicians. However, owing to the double-blind nature of the registrational studies, it was not possible to assess how physicians evaluate and trade off different product attributes (e.g. efficacy, safety, mode of administration). In physician-based studies conducted in North America, the potential to delay SREs was among the top attributes of BTAs considered by physicians, more so than risk of ONJ and method of treatment administration, when choosing therapy [26, 27]. To date, no studies have been conducted to assess European physicians’ preferences for any of the available BTAs.

The primary objective of this study was to quantify preferences for BTAs used for the prevention of SREs in patients with bone metastases secondary to solid tumours among treating physicians in France, Germany and the United Kingdom (UK).

## Methods

### Study sample

Physicians were eligible to participate in this study if they were currently involved in treating patients with bone metastases secondary to solid tumours. A research company (Harris Interactive) was engaged to recruit physicians from pre-existing physician panels in France, Germany and the UK between January and February 2013. These countries were selected as they are representative of major European markets and because the panels were already well established. Once physicians had been recruited and had provided informed consent, they were asked to complete a 25-min online discrete-choice experiment survey in their native language (i.e. English, French or German). In addition to the choice questions, basic physician demographic data were collected along with information about current level of experience in treating patients with bone metastases. These data were collated to aid interpretation of the results. Physicians were given the equivalent of approximately US$65 for participating in the study, but otherwise had no involvement with the study sponsor.

### Discrete-choice experiments

Discrete-choice experiments offer a systematic method of eliciting acceptable trade-offs in order to quantify the relative importance that respondants place upon various characteristics associated with hypothetical treatment options [27–29]. This approach is based on the premise that all treatments are composed of a set of key attributes (e.g. efficacy, safety, mode of administration) and the relative value an individual places on a particular treatment is therefore a function of these attributes [30, 31].

### Study design

The online discrete-choice experiment was developed and administered following current good research practice [32] for eliciting physicians’ treatment preferences. Treatment attributes were selected after reviewing product label information, scientific literature and clinical trial results, and after consultation with clinical experts regarding the relevant available BTAs including denosumab, zoledronic acid, clodronate and pamidronate [18–20, 25, 26, 33–37]. The attributes included: time until the first SRE; time until worsening of pain (at least a 2-point increase in the Brief Pain Inventory [BPI] scale); the annual risk of developing ONJ; the risk of renal impairment (defined as an annual risk of a 0.5 mg/dL increase in baseline serum creatinine level); and the mode of administration (Table 1). The levels of each attribute were chosen to encompass the range observed in current clinical practice as well as the range over which physicians are willing to accept trade-offs among attributes when evaluating hypothetical treatments.

**Table 1:** Attributes and levels for the choice questions

Attribute	Levels
Time until first SRE	28 months
	18 months
	10 months
Time until a 2-point increase in pain on the BPI	10 months
	6 months
	3 months
Risk of ONJ each year	None
	1 out of 100 (1%)
	5 out of 100 (5%)
Risk of 0.5 mg/dL increase in baseline creatinine level each year (risk of renal impairment)	None
	4 out of 100 (4%)
	10 out of 100 (10%)
Mode of administration	Daily oral tablet Injection every 4 weeks
	15-min infusion every 4 weeks
	120-min infusion every 4 weeks

BPI, Brief Pain Inventory; ONJ, osteonecrosis of the jaw; SRE, skeletal-related event

Prior to implementation, the attributes and chosen levels were validated through open-ended interviews with a test group of eight physicians who were currently treating patients with bone metastases in the United States of America (USA), where a similar discrete-choice experiment was conducted [26]. The study reported here was identical to the US study, with two exceptions. Out-of-pocket costs were not considered in this study because the treatments are partially or fully reimbursed in the EU countries included. Pamidronate and clodronate were not included in the US study because these agents are not approved (clodronate and oral pamidronate) or are rarely used (intravenous pamidronate) for the prevention of SREs in the US; consequently, daily oral tablet was not included as a method of administration in the US study. The interviews were conducted to assess the clarity and appropriateness of the descriptive information; to confirm that the five attributes included in the survey were salient to physicians and that no attributes were missing; and to determine whether the hypothetical patient profiles were representative of patients seen in their daily practice. Additional face-to-face interviews were conducted with a sample of physicians from the participating European countries (four in France, four in Germany and three in the UK) in order to test translation and cultural relevance. Data from these interviews were not included in the analysis as they were obtained solely for validation purposes.

**Fig. 1:**
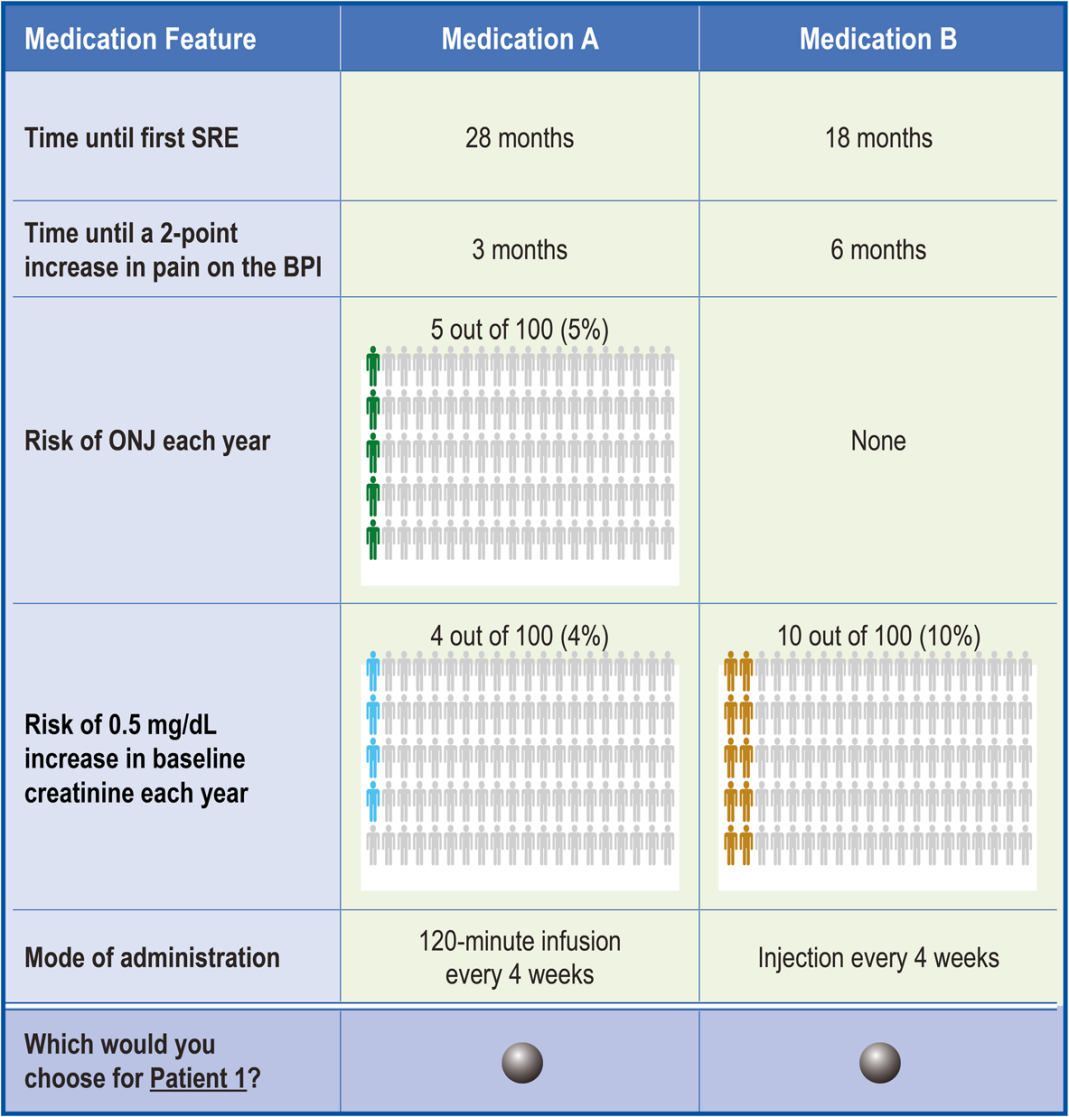
Patient 1 : A 57-year old woman who was diagnosed with breast cancer and developed bone metastases along with 2cm mediastinal and supraclavicular adenopathy 3 years after her initial diagnosis. She initially received treatment with docetaxel and cyclophosphamide adjuvant chemotherapy. The tumour is oestrogen receptor/progesterone receptor positive and HER-2 negative. She was on an adjuvant aromatase inhibitor at the time of her relapse. Her recurrence was noted by examination identifying the supraclavicular adenopathy. On further questioning, she admits to increasing mid-back (thoracic area) pain, which she rates as a 4 on a scale of 0 to 10*. The patient’s health is otherwise good (high performance status†) with no history of kidney disease and no significant comorbidities. Patient 2 : A 71-year-old man who was initially diagnosed with Gleason 8-10 prostate cancer 3 years ago. He is now castration-resistant and has developed bone metastases. His PSA level is ≥10. He is complaining of left hip pain when he walks and low back pain if he sits too long, which he rates as a 4 on a scale from 0–10*. The patient’s health is otherwise good (high performance status†) with no history of kidney disease and no significant comorbidities. *Where 0 is no pain and 10 is worst pain imaginable; †Karnofsky performance status. BPI, Brief Pain Inventory; HER-2, human epidermal growth factor-2; ONJ, osteonecrosis of the jaw; PSA prostate-specific antigen; SRE, skeletal-related event

An algorithm was used to develop hypothetical treatment profile pairs for the choice question sets. This algorithm was created using Statistical Analysis System (SAS) software Version 9.3 in order to construct a main-effects experimental design maximising D-efficiency. Using this methodology, an experimental design of 36 choice questions was developed and the design ensured that preferences for all attribute-level combinations were statistically identifiable. To avoid participant fatigue, the experimental design was divided into four versions, each with nine questions. These nine questions were then randomly ordered within each survey version. The third choice question from the four versions of choice questions was repeated as the seventh, eighth, or ninth choice question for internal validation (equating to a total of 10 choice questions per version; these responses were also included in the analysis). Each participating physician was then randomly assigned to receive one of the four questions sets and answered 10 choice questions in total. Because physicians may see patients at all stages of the disease, two profiles of what might be considered to be a typical patient with breast or prostate cancer were provided. Physicians were then asked to make their hypothetical treatment decisions based on these profiles (Fig. 1). Physicians reviewed identical patients’ profiles in all the countries.

Both the questions and the study protocol were reviewed by the Office of Research Protection and Ethics at RTI International (the responsible study organisation) and were approved by its institutional review board (IRB).

We used the test proposed by Swait and Louviere (1993) [38] to determine whether it was possible to pool the data from the three countries. In summary, we first created separate models for each country. We then constructed one pooled model in which we assumed that both preferences and error variances (also known as scale) were the same across countries and one model in which preferences were assumed to be homogeneous across countries, but scale was permitted to vary across countries. We used multinomial logit models and random parameters logit (RPL) models in our estimations and in both cases we tested (by mean of the Chow test, as suggested by Swait and Louviere) the hypothesis that the three countries have the same preferences. The hypothesis was rejected in both cases, thus a separate analysis was used for each country.

### Statistical analysis

RPL models [39, 40] were used to analyse responses to choice questions and quantify trade-off preferences among physicians in each country. The model results therefore reflect the effect of attribute levels on the likelihood that treatment A or B is selected. The parameter estimates from RPLs can be interpreted as the relative strength of preference for each attribute level, with more preferred outcomes having higher preference weights.

The 95% confidence interval (CI) was calculated and reported for each preference weight. If adjacent levels of a single attribute did not overlap, the mean estimates were considered to be statistically different from each other at the 5% level of significance.

The RPL model was also used to estimate predicted choice probabilities for any treatment profiles of interest, including profiles similar to actual treatment options.

Prediction of the proportion of physicians who would choose each profile can be calculated by applying the preference weights to the attribute levels included in the profile. Here, we report the predicted choice probabilities for physicans who would select a treatment profile with characteristics similar to: denosumab, zoledronic acid, clodronate and pamidronate (Table 2). Other available products (i.e. ibandronate) are not specified as their attributes fall within the range of the parameters for the products included.

**Table 2:** Treatment profiles and corresponding predicted choice probabilities

Attribute	Characteristics similar to denosumab	Characteristics similar to zoledronic acid	Characteristics similar to clodronate	Characteristics similar to pamidronate
Time until first SRE, months	27.7	19.5	15–20 (assume 17.5)	10.9
Time until worsening of pain, months	5.9	5.6	3.0	0.03–several (assume 3.0)
Risk of ONJ each year, %	1.8	1.3	Yes, but value not stated (assume 1.0)	Yes, but value not stated (assume 1.0)
Risk of renal impairment each year, %	0	9.3	Yes, but value not stated (assume 5.0)	8.1
Mode of administration	Injection every 4 weeks	15-min infusion every 4 weeks	Daily oral tablet	120-min infusion every 4 weeks
Predicted choice probabilities; country, mean (95% CI)				
France	90.4 (84.1, 94.2)	3.9 (2.0, 7.1)	5.3 (3.1, 9.0)	0.4 (0.1, 1.0)
Germany	93.5 (88.9, 96.3)	3.6 (1.9, 6.7)	2.6 (1.5, 4.5)	0.2 (0.1, 0.6)
UK	90.3 (84.8, 94.0)	3.8 (2.1, 6.6)	5.6 (3.3, 9.0)	0.3 (0.1, 0.7)

Values for pain worsening for denosumab and zoledronic acid were based on von Moos et al. 2013 [25], and for clodronate on Jagdev et al. 2001 [36]. The value for time until complication of bone metastases for clodronate was based on Kristensen et al. 1999 [37]. All other values were derived from the prescribing information for denosumab, zoledronic acid, clodronate and pamidronate [18–20, 33–35], with assumptions made, as stated, where definitive published values were absent

CI, confidence interval; ONJ, osteonecrosis of the jaw; SRE, skeletal-related event; UK, United Kingdom

## Results

### Participants

A total of 3553 physicians in the UK, 3872 in France and 1746 in Germany who were currently treating patients with bone metastases were invited to participate in the online discrete-choice experiment. Of those invited, 324 physicians in the UK, 330 in France and 304 in Germany responded to the invitation. Of those physicians who responded, 241 physicians in the UK, 245 in France and 238 in Germany were eligible and 236, 241 and 233 physicians in each country, respectively, consented to participate. Of the consenting physicians, 200 physicians in each country completed the survey as per the quota sampling approach (i.e. at least 150 physicians in each country).

Of the eligible physicans, three physicians from the UK, nine physicians from France and eight physicians from Germany were excluded from the analysis because they chose the same answer throughout the entire set of questions (i.e. always selecting Treatment A or Treatment B). This indicated a lack of attention to the questions and thus their responses were not included in the final analysis. The final sample included responses from 197 physicians in the UK, 191 physicians in France and 192 physicians in Germany.

The demographic characteristics of the participating physicians are listed in Table 3. In all three countries, the majority of physicians had been practising medicine for more than 10 years since completing their medical training. In both the UK and France, the majority of the participating physicians indicated that they were working in national health service hospitals (98.0 and 73.8%, respectively). In Germany, only 30.2% of the physicians were working for the state healthcare system and another 38.5% worked in office-based private practices. Across all three countries the primary area of speciality of the participating physicians was oncology. As required by the eligibility criteria, all physicians were currently treating patients with bone metastases from solid tumours.

**Table 3:** Demographic characteristics of participating physicians

Category	n (%)		
	UK (*n* = 197)	France (*n* = 191)	Germany (*n* = 192)
Age, years			
18–45	129 (65.5)	113 (59.2)	82 (42.7)
46–75	68 (34.5)	78 (40.8)	110 (57.3)
Table 1. How many years have you been in practice since completing your medical training?			
< 10	34 (17.2)	61 (32.1)	32 (16.7)
≥10	163 (82.8)	129 (67.9)	160 (83.3)
Which of the following best describes your area of specialty?			
Primary care	6 (3.1)	5 (2.6)	10 (5.2)
Family medicine	2 (1.0)	0	1 (0.5)
Oncology	97 (50.0)	118 (62.4)	91 (47.4)
Other	89 (45.9)	66 (34.9)	90 (46.9)
On average, how many patients with bone metastases from solid tumours do you treat each week?			
≤10	129 (65.5)	115 (60.2)	131 (68.2)
> 10	68 (34.5)	76 (39.8)	61 (31.8)

Counts and percentages displayed exclude missing values and may not add up to the total country sample size or 100%

UK, United Kingdom

### Preference weights

Tables 4a–c present estimated preference weights for all attribute levels for physicians in France, Germany and the UK. The scaled relative importance estimates for the attributes are the vertical distances that individual attributes span/cover. The longer the distance for the attribute, the greater the importance of that attribute. For all three countries, time until the first SRE was considered by physicians to be relatively the most important attribute and risk of ONJ each year was considered to be relatively the least important attribute (Table 5). There was little congruence between the countries regarding the relative ordering of the remaining attributes (time until pain worsening, risk of renal impairment and mode of administration). However, as noted above, the study was not designed to evaluate whether there were statistically significant differences between countries.

**Table 4:** Physician preference weights for (a) France; (b) Germany; (c) UK

Attribute Name	Level	Preference weight (95% CI)	*p* value
(a) France			
Time until first SRE	28 months	1.44 (1.15, 1.72)	0.000
	18 months	− 0.13 (− 0.26, 0.01)	0.068
	10 months	−1.31 (− 1.60, − 1.02)	0.000
Time until a 2-point increase in pain on the BPI	10 months	0.36 (0.18, 0.54)	0.000
	6 months	0.18 (0.03, 0.33)	0.019
	3 months	−0.54 (− 0.72, − 0.36)	0.000
Risk of ONJ each year	None	0.15 (−0.02, 0.31)	0.086
	1 out of 100 (1%)	0.22 (0.07, 0.37)	0.005
	5 out of 100 (5%)	−0.37 (−0.54, − 0.19)	0.000
Risk of 0.5 mg/dL increase in baseline creatinine each year	None	0.93 (0.68, 1.17)	0.000
	4 out of 100 (4%)	−0.07 (−0.23, 0.10)	0.427
	10 out of 100 (10%)	−0.86 (−1.09, − 0.64)	0.000
Mode of administration	Daily oral tablet	0.57 (0.35, 0.79)	0.000
	Injection every 4 weeks	0.12 (−0.08, 0.31)	0.239
	15-min infusion every 4 weeks	−0.04 (− 0.24, 0.16)	0.677
	120-min infusion every 4 weeks	−0.64 (− 0.88, 0.40)	0.000
(b) Germany			
Time until first SRE	28 months	1.48 (1.19, 1.78)	0.000
	18 months	−0.31 (−0.47, − 0.16)	0.000
	10 months	−1.17 (−1.46, −0.88)	0.000
Time until a 2-point increase in pain on the BPI	10 months	0.98 (0.70, 1.25)	0.000
	6 months	0.04 (−0.12, 0.19)	0.649
	3 months	−1.01 (−1.19, −0.83)	0.000
Risk of ONJ each year	None	0.40 (0.22, 0.58)	0.000
	1 out of 100 (1%)	0.27 (0.11, 0.44)	0.001
	5 out of 100 (5%)	−0.67 (−0.85, −0.50)	0.000
Risk of 0.5 mg/dL increase in baseline creatinine each year	None	0.80 (0.58, 1.01)	0.000
	4 out of 100 (4%)	0.11 (−0.08, 0.29)	0.264
	10 out of 100 (10%)	−0.90 (−1.13, − 0.68)	0.000
Mode of administration	Daily oral tablet	0.30 (0.08, 0.52)	0.008
	Injection every 4 weeks	0.41 (0.20, 0.63)	0.000
	15-min infusion every 4 weeks	0.21 (0.01, 0.41)	0.042
	120-min infusion every 4 weeks	−0.92 (−1.16, − 0.68)	0.000
(c) UK			
Time until first SRE	28 months	1.25 (1.00, 1.51)	0.000
	18 months	−0.08 (−0.21, 0.06)	0.275
	10 months	−1.18 (−1.43, −0.92)	0.000
Time until a 2-point increase in pain on the BPI	10 months	0.80 (0.59, 1.01)	0.000
	6 months	0.17 (0.02, 0.32)	0.028
	3 months	−0.97 (−1.18, − 0.76)	0.000
Risk of ONJ each year	None	0.28 (0.13, 0.43)	0.000
	1 out of 100 (1%)	0.37 (0.21, 0.53)	0.000
	5 out of 100 (5%)	−0.64 (−0.83, − 0.46)	0.000
Risk of 0.5 mg/dL increase in baseline creatinine each year	None	0.89 (0.68, 1.10)	0.000
	4 out of 100 (4%)	0.07 (−0.10, 0.23)	0.434
	10 out of 100 (10%)	−0.96 (−1.17, − 0.74)	0.000
Mode of administration	Daily oral tablet	0.69 (0.47, 0.92)	0.000
	Injection every 4 weeks	0.23 (0.05, 0.41)	0.011
	15-min infusion every 4 weeks	−0.13 (− 0.31, 0.06)	0.175
	120-min infusion every 4 weeks	−0.79 (−1.02, − 0.57)	0.000

BPI, Brief Pain Inventory; CI, confidence interval; ONJ, osteonecrosis of the jaw; SRE, skeletal-related event

**Table 5:** Relative importance of attributes in decreasing order

Relative importance	UK	France	Germany
1	Time until first SRE	Time until first SRE	Time until first SRE
2	Risk of renal impairment each year	Risk of renal impairment each year	Time until worsening of pain
3	Time until worsening of pain	Mode of administration	Risk of renal impairment each year
4	Mode of administration	Time until worsening of pain	Mode of administration
5	Risk of ONJ each year	Risk of ONJ each year	Risk of ONJ each year

ONJ, osteonecrosis of the jaw; SRE, skeletal-related events; UK, United Kingdom

Within each attribute, among physicians from France and the UK, preference weights followed the predicted ordering based on the severity of outcomes for all the applicable numerical attributes levels, except for no risk and 1% risk of ONJ, for which physicians did not perceive there to be a difference between the two levels (Tables 4a and c). In Germany (Table 4b), preference weights followed the natural ordering for all attributes. The preference weights for all levels were statistically significantly different from each other (*p* < 0.05) for time until the first SRE and risk of renal impairment in all countries (Table 4). The preference weights for all levels of time until worsening of pain were also statistically significantly different from each other in both the UK and Germany (*p* < 0.05). In relation to the mode of administration, for the UK, preference for a daily oral tablet, a subcutaneous injection, a 15-min infusion and a 120-min infusion were statistically significantly different from each other (*p* < 0.05). In France, preference for a subcutaneous injection and a 15-min infusion were not statistically significantly different from each other (*p* > 0.05). In Germany, preference for a daily oral tablet, a subcutaneous injection and a 15-min infusion were not statistically significantly different from each other (*p* > 0.05). Across all three countries, the 120-min infusion every 4 weeks was the least preferred mode of administration.

Predicted choice probabilities in terms of the likelihood that one treatment with its associated attributes would be chosen over the other treatment are detailed in Table 2. Physicians were not directly asked about their preference for existing treatment options. Instead, their preferences for a particular treatment were estimated by adding the preference weights (Table 4) for the attribute levels included in individual treatment profile outlined in Table 2, and this was a totality of physicians’ preferences regarding SRE prevention, delay in pain worsening, risk of ONJ, risk of renal impairment and mode of administration. It was estimated that in all three countries, the majority of physicians preferred a treatment with attributes similar to denosumab (90.3% in the UK, 90.4% in France and 93.5% in Germany).

## Discussion

The study findings suggest that the relative efficacy of the BTA in delaying SREs and its potential for causing renal impairment play a relatively greater role when physicians are forming treatment decisions compared with other aspects associated with treatment, such as the potential risk of ONJ. In our study, there was little or no difference across all countries in terms of physicians’ preference between no risk of ONJ and a 1% annual risk of ONJ. This may be because physicians are increasingly aware of good dental health steps that can be taken before and during treatment with a BTA in order to reduce the risk of this complication. Some authors have claimed in recent publications that, through patient and healthcare education, proactive monitoring and early diagnosis, ONJ management is now focused on conservative approaches [41–43]. Thus, our data likely reflect the fact that physicians widely accept the positive risk–benefit profile associated with BTAs.

From the perspective of the treating physicians, limited importance was placed on the mode of administration of the BTA. In addition to concern over the patient’s burden of treatment administration (requiring insertion of an intravenous line or remaining upright for a period of time after taking an oral tablet), physician preference for one type of mode of administration over another may be driven by other aspects such as reimbursement incentives for intravenous administration, availability of an intravenous infusion chair, concerns regarding patient adherence/persistence with oral treatments or the desire to see the patient on a regular basis.

Delaying SREs and avoiding renal impairment were also selected as the most important clinical attributes from a physician’s perspective in the US discrete-choice study and in a similar experiment in Canada and, in line with the results of our study, less importance was placed on treatment administration and the risk of ONJ [26, 27]. These data confirm that treatments that delay SREs and carry a low risk of renal impairment are desirable in the opinions of physicians from both Europe and North America.

While it is not uncommon to observe discrepancies between physicians’ and patients’ preferences, overall, the findings from this study appear to be in concordance with findings from a similarly designed, patient focused discrete-choice experiment in which self reported physican diagnosed patients from online panels were asked to evaluate hypothetical treatment profiles [44]. Consenting patient participants were asked to select from hypothetical Treatment A or Treatment B derived from the prescribing information of currently available BTAs, with the same attributes as those listed for physicians. For patients, the most important characteristics when selecting treatments were generally delaying SREs, avoiding renal impairment and delaying pain worsening, while risk of ONJ was the least important.

Based on the results of the predicted choice probability analysis, physicians preferred a hypothetical treatment profile with attributes similar to denosumab. This is driven by their desire to delay the symptoms of SREs, to manage the annual risk of renal impairment (historically associated with bisphosphonates) and to prevent the worsening of pain.

The self-selection of paticipants (from the wider invitee population) is acknowledged as a study limitation and source of potential bias. Willingness to participate may reflect some inherent bias in the profile (charateristics/traits) of the participating physicians (e.g. age, professional experience, caseload, setting) and may limit the generalisability of the results to a wider physician population. No data on the representativeness of the participating physician are available to qualify the extent of this potential bias. Discrete-choice experiments also have an inherent number of limitations. First, preference is inferred based on choices of hypothetical treatment profiles from the survey instrument. In order to minimise the hypothetical bias, we attempted to make the hypothetical choices mimic real-world trade-offs as closely as possible and verified the attributes included in the survey with clinical experts in the open-ended interviews during survey testing. Nevertheless, values assigned to the attribute levels could influence the degree of importance placed on each attribute by physicians. For example, zoledronic acid can be given as frequently as every 3 weeks; however, data suggest that the vast majority (83%) of patients receive zoledronic acid every 4 weeks [45], and so we did not include administration every 3 weeks as an option. Furthermore, hypothetical choices are based on the ‘ideal’ whereas, in reality, the treatment option with the corresponding treatment attributes may not be reimbursed for use in a particular country. As such, we did not determine whether or not costs and current reimbursement situations would further influence physicians’ decisions. It is, however, likely that the financial implications of treatment decisions for healthcare systems influence clinical practice.

## Conclusion

Our discrete-choice experiment found that, in France and the UK, time until the first SRE and risk of renal impairment were the most important attributes when choosing a BTA, whereas in Germany, time until the first SRE and delay in worsening of pain were the most important. It was estimated that the majority of physicians in all three countries preferred a BTA with attributes that were most similar to those of denosumab. To the best of our knowledge, this is the first study to quantify European physicians’ preferences for attributes when choosing a BTA for the active prevention of SREs caused by bone metastases arising in patients with solid tumours. Such an assessment of available treatment options provides insight into the physicians’ treatment decision making and may further help to inform treatment practice, assist in reimbursement decisions and improve overall disease and patient management.

## Abbreviations

BPI: Brief Pain Inventory
BTA: Bone-targeted agent
CI: Confidence interval
EU: European Union
IRB: Institutional review board
ONJ: Osteonecrosis of the jaw
RANK: Receptor activator of nuclear factor kappa B
RPL: Random parameters logit
SAS: Statistical analysis system
SRE: Skeletal-related event
UK: United Kingdom
USA: United States of America
